# Supplementary material for: Experience of Euclidean geometry sculpts the development and dynamics of rodent hippocampal sequential cell assemblies
Source: Nat Commun. 2024 Sep 28;15:8417. doi: 10.1038/s41467-024-52758-9 (PMC11438871; doi:10.1038/s41467-024-52758-9)
Supplement: Supplementary file 1 — Supplementary Information [file 41467_2024_52758_MOESM1_ESM.pdf]

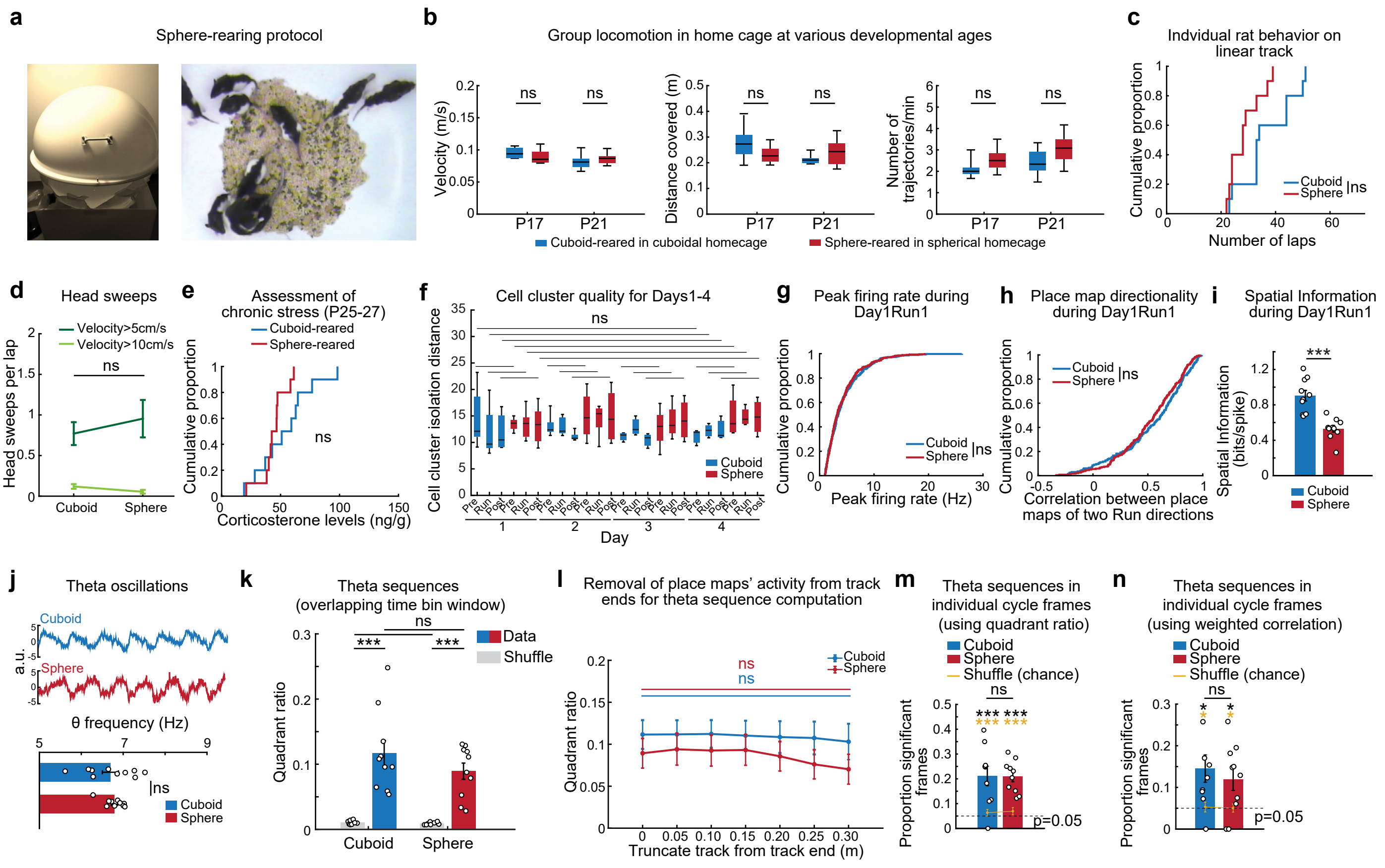

Supplementary Fig. 1: Rat behavior in corresponding home cages during the third postnatal week and on the linear track during Day1Run1, neuronal clustering quality of recorded units, single-cell properties, and theta oscillations in cuboid- and sphere-reared rats. a Photo of a sphere used for the sphere-rearing protocol (left) and a top-view camera snapshot of a P21 rat litter with the dam inside a sphere (right). The rat pups forayed beyond the bedding interacting with the geometric curvature. **b** Assessment of behavior of whole litter in cuboidal and spherical home cages at P17 and P21. Note similar linearized velocity in trajectories (left, p=0.28 and p=0.40, respectively), distance covered in trajectories (center, p=0.19 and p=0.34, respectively) and number of trajectories per minute (right, p=0.10 and p=0.11, respectively) in cuboidal and spherical home cages at P17 and P21 (two-sided rank-sum tests, N=7/8 cuboid/sphere-reared rat pups). **c-d** Similarity of rat behavior on the linear track between cuboid- and sphere-reared groups on Day1Run1. Number of laps (>65% of track length/lap) during Day1Run1 (c) were not different between groups (p=0.062, two-sided rank-sum test). Lateral head sweeps/lap (d) at different thresholds of velocity (5cm/s and 10cm/s) were similar on Day1Run1 (p=0.50 and p=0.092 respectively, two-sided t-tests). **e** Similar corticosterone levels in fecal pellets collected during exploration of linear tracks of cuboid- and sphere-reared rats at P25-27 (p=0.26, two-sided t-test, N=10 rats/group). **f** Cluster isolation distance for recorded units for various recording sessions (Pre-Run sleep, Run sessions, Post-Run sleep), groups (cuboid- vs. sphere-reared) and days of experiment (Days 1, 2, 3 and 4). Kruskal-Wallis ANOVA tests (for >2 groups) or rank-sum tests (for pairs of groups) were conducted to study the effect of each of these factors on cluster quality (cuboid Pre/Run/Post across days: p=0.33/p=0.74/p=0.63, sphere Pre/Run/Post across days: p=0.90/p=0.79/p=0.90, cuboid Pre vs. Run vs. Post: p=0.17, sphere Pre vs. Run vs. Post: p=0.97, ANOVAs, cuboid vs. sphere Pre Day1-4: p=0.84, 0.69, p=0.4, p=0.4, cuboid vs. sphere Run Day1-4: p=0.42, 0.31, p=0.86, p=0.11, cuboid vs. sphere Post Day1-4: p=0.69, 0.41, p=0.23, p=0.4, two-sided rank-sum tests). **g** Peak firing rate of sphere- and cuboid-reared place cells was similar during Day1Run1 (p=0.60, two-sided rank-sum test). **h** Sphere-reared rats exhibited normal development of place map similarity for the two run directions at P24 (cuboid vs. sphere, p=0.20, two-sided rank-sum test). **i** Spatial information of place cells in P24 sphere-reared rats was lower than in P23-24 controls (p=0.00044, two-sided rank-sum test) during Day1Run1. **j** The peak frequency of theta oscillation during locomotion was similar in cuboid- and sphere-reared rats (p=0.91, two-sided rank-sum test at velocity>10 cm/s). **k** Theta sequences compared across groups (p=0.25, two-sided t-test) and against shuffles (p=2.1x10^{-4}, p=6.0x10^{-5}, paired one-sided t-tests) using the quadrant ratio method and an overlapping time bin window. **l** Increasingly truncating the track by removing place map activity at track ends did not significantly impact theta sequences which remained similar across groups (truncation: 0 to 0.30 m: cuboid vs. sphere: p=0.38, p=0.49, p=0.44, p=0.51, p=0.39, p=0.25, p=0.25, two-sided t-tests; 0 vs 30 m, cuboid: p=0.15, sphere: p=0.11, paired two-sided t-tests), using the quadrant ratio method and a non-overlapping time bin window. **m-n** Theta sequences compared across groups (p=0.95, p=0.56, two-sided t-tests) , against shuffle (p=0.0015, p=7.1x10^{-5}, p=0.012, p=0.012, one-sided t-tests, yellow stars), and against p=0.05 chance (p=0.0022, p=1.4x10^{-5}, p=0.012, p=0.014, one-sided t-tests, black stars; p=0, one-sided Binomial tests vs. p=0.05 chance for both groups) using a non-overlapping time bin window and the quadrant ratio method (m) or weighted correlation method at the individual theta cycle frame level (n). (c-d, g-n), groups are P24 sphere-reared and P23-24 cuboid-reared rats on Day1 of the experiment. N=10 (5 rats/group; 2 directions/rat). Data are displayed as mean±standard error of the mean. ***p<0.005. ns=not significant.

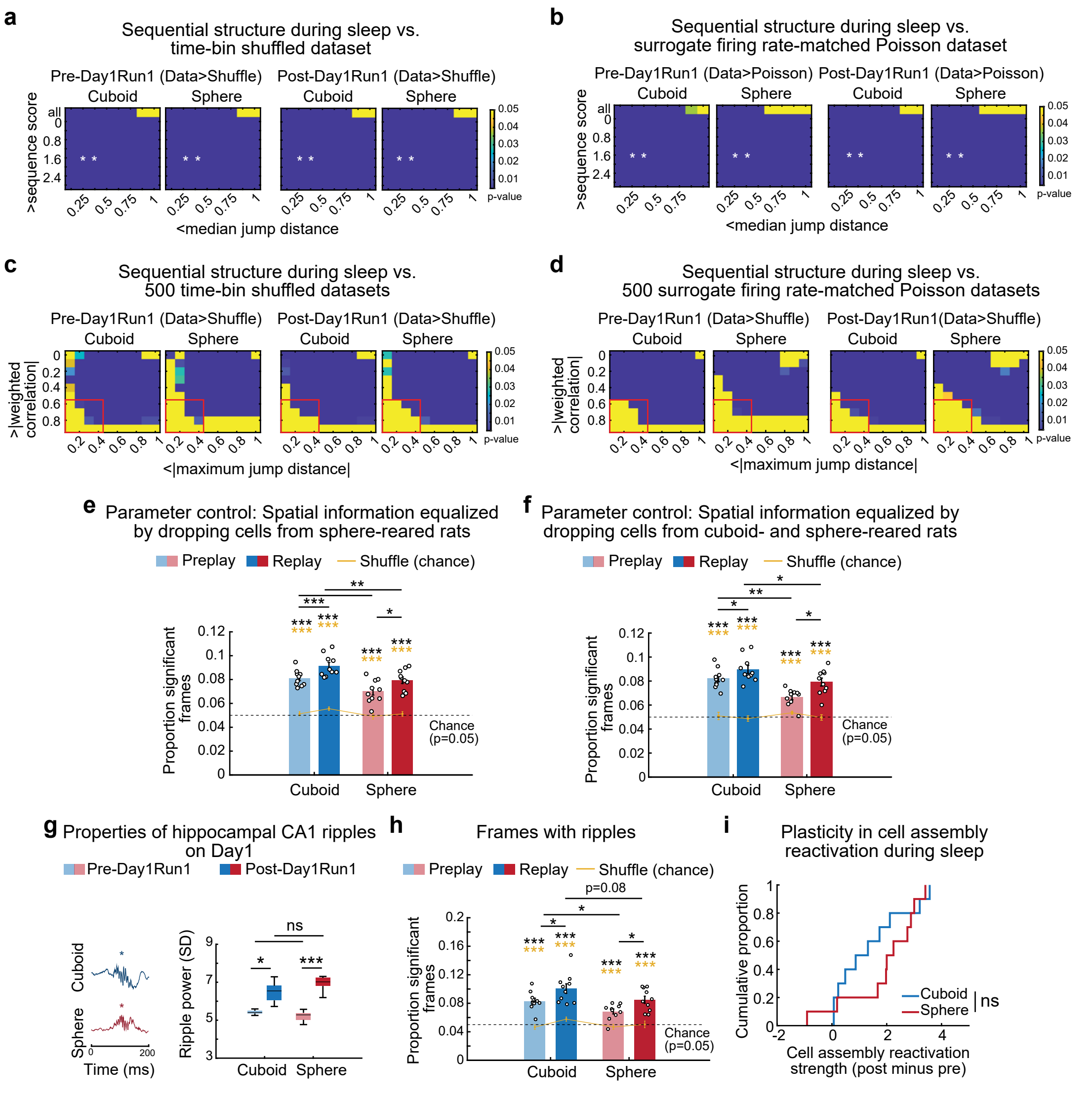

Supplementary Fig. 2: Individual place cell properties do not account for the variability in trajectory sequences across groups on Day1 in sphere-reared rats. a Cuboid- and sphere-reared rats exhibited higher sequential structure (as assessed by simultaneous 2-feature - sequence score and median jump distance - comparisons of trajectory sequences) than within-frame time-bin shuffled datasets in the Pre-Day1Run1 and Post-Day1Run1 sleep sessions flanking the de novo run. Stars indicate comparisons at relevant parameter thresholds (i.e., sequence scores>1.6 and median jump distances<0.375, 0.25). **b** Cuboid- and sphere-reared rats exhibited higher sequential structure (as assessed by simultaneous 2-feature - sequence score and median jump distance - comparisons of trajectory sequences) than the firing rate-matched corresponding surrogate Poisson datasets in the Pre-Day1Run1 and Post-Day1Run1 sleep sessions flanking the de novo run. Stars indicate comparisons at relevant parameter thresholds (i.e., sequence scores>1.6 and median jump distances<0.375, 0.25). **c** Cuboid- and sphere-reared rats exhibited higher sequential structure (as assessed by simultaneous 2-feature - absolute weighted correlation and maximum jump distance - comparisons of trajectory sequences) than the within-frame time-bin shuffled datasets in the Pre-Day1Run1 and Post-Day1Run1 sleep sessions flanking the de novo run. Red rectangle depicts sets of thresholds with better sequential content as assessed by these features. **d** Cuboid- and sphere-reared rats exhibited higher sequential structure (as assessed by simultaneous 2-feature - absolute weighted correlation and maximum jump distance - comparisons of trajectory sequences) than the firing rate-matched corresponding surrogate Poisson datasets in the Pre-Day1Run1 and Post-Day1Run1 sleep sessions flanking the de novo run. Red rectangle depicts sets of thresholds with better sequential content as assessed by these features. **e** Differences in spatial information of neurons did not contribute to the observed group differences in trajectory sequences. By dropping cells with low spatial information in sphere-reared rats until spatial information became similar across both groups, we studied the proportion of significant preplay frames, replay frames and plasticity in replay. A higher proportion of frames depicted trajectory sequences in Pre-/Post-Day1Run1 sleeps in cuboid- compared to sphere-reared rats (p=0.0079/p=0.0083, two-sided t-tests). However, sphere-reared rats depicted robust trajectory sequences, as studied by the proportion of significant frames (sphere Pre-/Post- vs. shuffle: p=4.2x10^{-4}, p=4.0x10^{-5}, paired one-sided t-tests, yellow stars; sphere Pre-/Post- vs. p=0.05 chance: p=5.0x10^{-5}, p=1.2x10^{-6}, t-tests, black stars; p=0, p=0, one-sided Binomial tests for Pre-/Post-Day1Run1 sleeps vs. p=0.05 chance). **f** Differences in spatial information of neurons did not contribute to the observed group differences in trajectory sequences. By dropping cells with high spatial information in cuboid- and low spatial information in sphere-reared rats until spatial information became similar across both groups, we studied the proportion of significant preplay frames, replay frames and plasticity in replay. A higher proportion of frames depicted trajectory sequences in Pre-/Post-Day1Run1 sleeps in cuboid- compared to sphere-reared rats (p=2.7x10^{-4}/p=0.037, two-sided t-tests). However, sphere-reared rats depicted robust trajectory sequences, as studied by the proportion of significant frames (sphere Pre-/Post- vs. shuffle: p=9.3x10^{-4}, p=2.2x10^{-6}, paired one-sided t-tests, yellow stars; sphere Pre-/Post- vs. p=0.05 chance: p=2.0x10^{-5}, p=4.8x10^{-6}, one-sided t-tests, black stars; p=0, one-sided Binomial tests for Pre-/Post-Day1Run1 sleeps vs. p=0.05 chance). **g** Examples of ripples in cuboid and sphere-reared rats during non-rapid-eye-movement Pre- and Post-Day1Run1 sleep sessions (1st column from left). Peak power of sleep ripples expressed as standard deviations above mean were similar (2nd column; cuboid vs. sphere-reared, p=0.18, p=0.19, t-tests for Pre- and Post-Day1Run1 sleep sessions). Both groups showed increases from Pre- to Post-Day1Run sleep (p=0.012, p=0.0031, respectively, paired two-sided t-tests). **h** Proportions of ripple-coincident frames with significant preplay, replay were above chance levels in the cuboid- and sphere-reared rats (cuboid Pre-/Post- vs. shuffle: p=2.0x10^{-6}, p=5.0x10^{-5}, sphere Pre-/Post- vs. shuffle: p=0.0015, p=6.5x10^{-4}, paired one-sided t-tests, yellow stars; cuboid Pre-/Post- vs. p=0.05 chance: p=1.1x10^{-5}, p=1.7x10^{-5}, sphere Pre-/Post- vs. p=0.05 chance: p=2.5x10^{-4}, p=3.0x10^{-5}, one-sided t-tests, black stars; p=0, p=0, p=2.3x10^{-8}, p=0, one-sided Binomial tests vs. p=0.05 chance for each group respectively). Preplay was at lower levels (p=0.015, two-sided t-test) while replay was tending towards being significantly lower compared with the cuboid-reared rats (p=0.076, two-sided t-test). **i** Cell-assembly reactivation during Post-Day1Run1 sleep. Plasticity in reactivation was similar in cuboid- and sphere-reared rats (p=0.34, two-sided rank-sum test). (a-i), N=10 (5 rats/group; 2 directions/rat). Data are displayed as mean±standard error of the mean. ***p<0.005. **p<0.01. *p<0.05. ns=not significant.

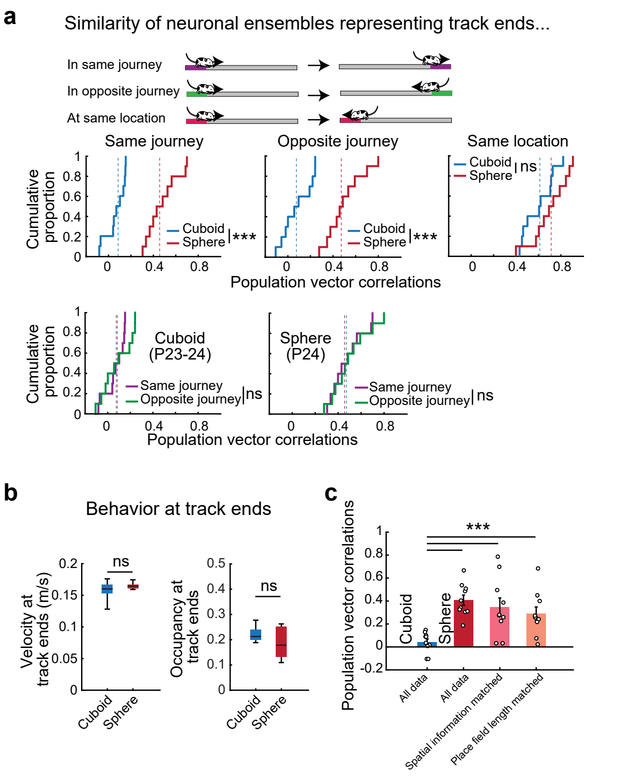

Supplementary Fig. 3: Detailed characterization of higher similarity of the neuronal ensemble representations of track ends in the sphere-reared rats. **a** Neuronal ensembles in sphere-reared rats represented track ends more similarly than cuboid-reared rats when the rat took the same direction journey (p=0.00018, two-sided rank-sum test), the opposite direction journey (p=0.00018, two-sided rank-sum test), but not at the same absolute location in space during Day1Run1 (p=0.19, two-sided rank-sum test). **b** Cuboid- and sphere-reared rats had similar velocity (left, p=0.27, two-sided rank-sum test) and occupancy (right, p=0.19, two-sided rank-sum test) at track ends. **c** Population vectors representing the two track ends had significantly higher similarity in sphere-reared rats (p=0.0046 or p=0.0036, two-sided rank-sum tests) in reduced datasets with matched spatial information or place field length with cuboid-reared rats. N (number of rats), Cuboid: 5 (P23-24), Sphere: 5 (P24). Data are displayed as mean±standard error of the mean. ***p<0.005. ns=not significant. Panel 3a cartoons adapted from U. Farooq, G. Dragoi, Emergence of preconfigured and plastic time-compressed sequences in early postnatal development. Science 363, 168-173 (2019). DOI: 10.1126/science.aav0502. Reprinted with permission from AAAS.

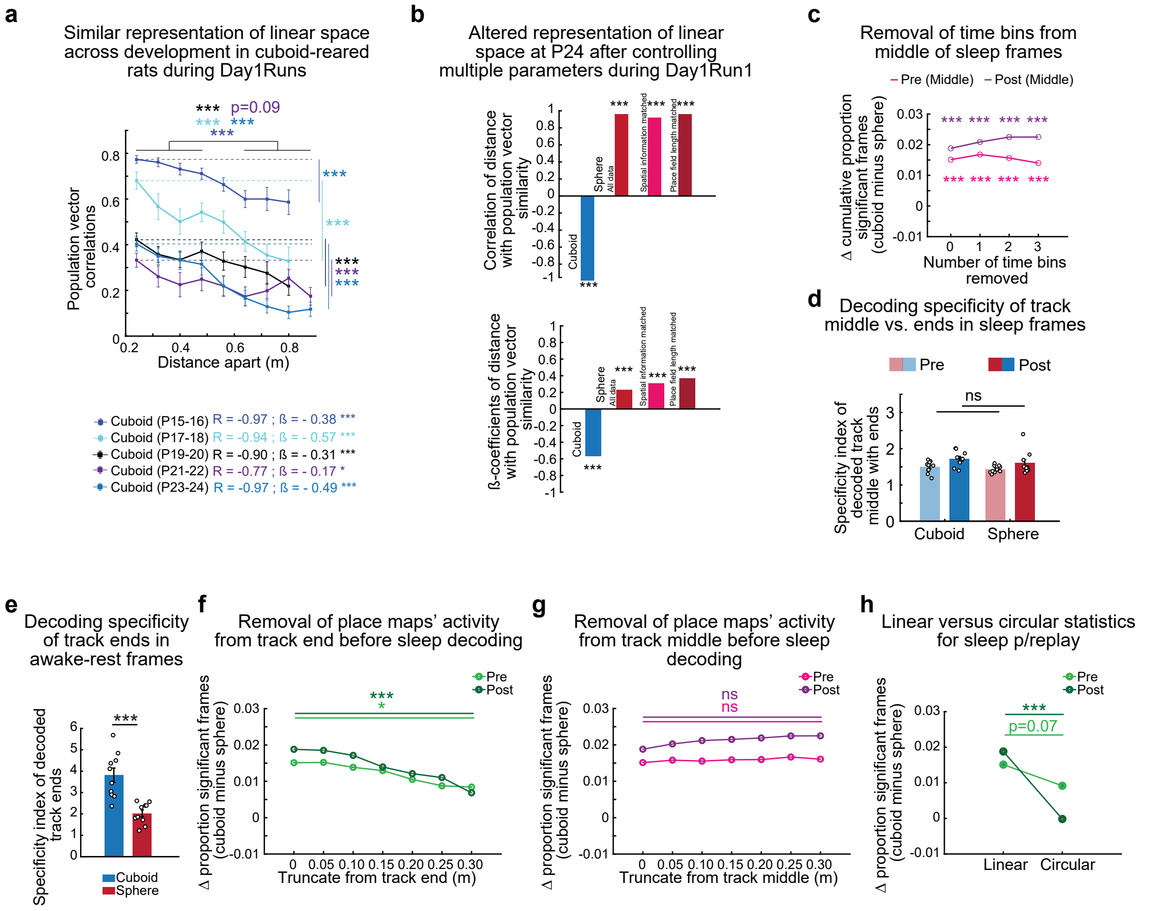

Supplementary Fig. 4: Changes in hippocampal ensemble depiction of linear space extends beyond track ends/corners after sphere-rearing. a Correlation and ß-coefficients of the linear regression fit between the length of distance apart on the linear track and the population vector similarity were negative at all ages across development in cuboid-reared rats during Day1Runs, and positive in the sphere-reared rats. Note a decrease at all developmental ages in population vector correlation with distance (significance depicted on top of plot, the P21-22 group was tending to significance, rank-sum tests), and on comparison of nearest and furthest points on the track (from P15-16 to P23-24: p=0.0014, p=4.3x10^{-4}, 1.6x10^{-4}, p=0.0033, p=2.1x10^{-6}, two-sided signed-rank tests), depicted by stars on right of the plot. N (rats/group x 2 directions/rat for each run session): Cuboid, P15-16 (10), P17-18 (6), P19-20 (8), P21-22 (8), P23-24 (10). **b** Pearson's correlation (left) and ß-coefficients (slope, right) of the linear regression relationship between distance apart on the linear track with population vector similarity remained significantly positive in the sphere-reared rats, unlike cuboid-reared rats, after restricting to place cells with matched spatial information and place field lengths to cuboid-reared rats. **c** Difference in cumulative proportion of significant frames between cuboid and sphere-reared rats in Pre-Day1Run1 and Post-Day1Run1 sleep on generation of surrogate data with removal of 1-3 20-ms time bins from the middle of the sleep frames. Removing time bins from the middle did not result in equalization of the cumulative proportion of significant frames in cuboid and sphere-reared rats in Pre- and Post-Day1Run1 sleep (Z-test for 2 proportions on pooled data across all animals). **d** Specificity index of decoded middle with track ends was similar across the two groups (Pre: p=0.34, Post: p=0.32, two-sided t-tests). **e** Specificity index of decoded track ends during awake-rest frames (p=0.00013, two-sided t-test), similar analysis to Fig. 3g for sleep. **f** Increasingly truncating the track by removing place map activity at the track ends significantly reduced the differences in proportion significant p/replay frames across groups (permutation tests for comparison of non-truncated and 0.30 m truncated data in cuboid- vs. sphere-reared rats). **g** Increasingly truncating the track by removing place map activity from the track middle did not significantly impact the differences in proportion significant p/replay frames across groups (permutation tests for comparison of non-truncated and 0.30 m truncated data in cuboid- vs. sphere-reared rats). **h** Usage of circular statistics to compute the decoded space-time weighted correlations assuming circular space reduced the differences between proportions of significant p/replay frames across groups (permutation tests for comparison of linear vs. circular statistics on pooled data for comparison of cuboid- vs. sphere-reared rats). (b-h) N=10 (Cuboid, P23-24 vs. Sphere, P24: 5 rats/group; 2 directions/rat). ***p<0.005. *p<0.05.

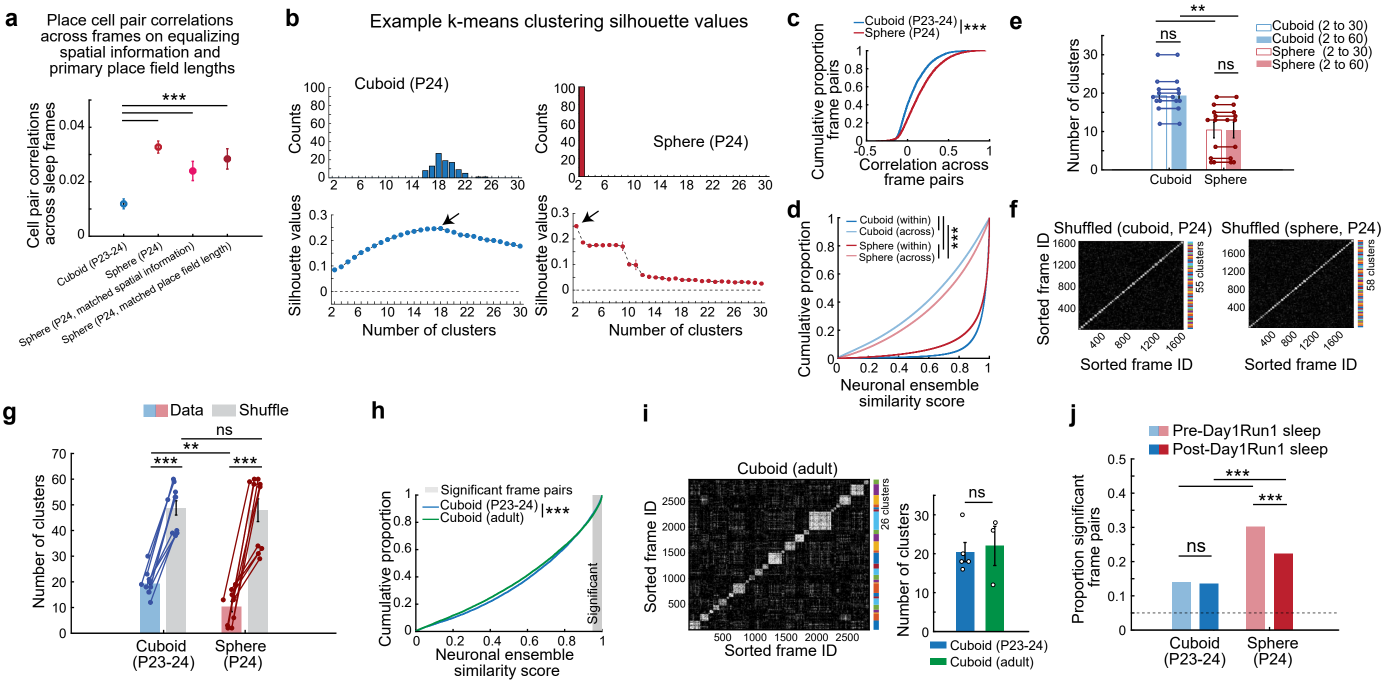

Supplementary Fig. 5: Properties of clusters of neuronal ensembles activated in sleep frames in experimentally-naïve cuboid- and sphere-reared rats. a Cell pair correlations across frames during sleep for place cells were significantly higher in sphere-reared rats ($p=4.4x10^{-11}$, two-sided rank-sum test) and remained significantly higher ($p=0.00036$ and $p=5.9x10^{-5}$, two-sided rank-sum tests) after restricting to place cells with matched spatial information and place field lengths to cuboid-reared rats. **b** (Bottom) Median silhouette values (100 iterations) for the detected k-means clusters in representative sleep neural data from a cuboid-reared (left) and a sphere-reared rat (right). (Top) Counts (out of 100 iterations) of number of clusters with maximal silhouette value for each iteration. Mode of number of clusters with maximum silhouette values was 18 clusters for the cuboid-reared (left) and 2 clusters for the sphere-reared rat (right). **c** Correlations of activated neuronal ensembles across frame pairs were significantly higher in sphere-reared rats ($p=2.2x10^{-284}$, two-sided rank-sum test). **d** Neuronal ensemble similarity scores of frame pairs within cluster were significantly higher than across clusters for both cuboid- and sphere-reared rats (p=0 and p=0, two-sided rank-sum tests). **e** Clustering results are robust to chosen range of the k-means clustering algorithm, tested at ranges 2 to 30 clusters and 2 to 60 clusters for Day1 sleeps in cuboid- and sphere-reared rats (p=1, p=1, two-sided signed-rank tests). Note no sleep session showed a difference between the two ranges. Number of clusters for individual sleeps (presented as dot markers connected by lines) were jittered on the x-axis to aid visualization of sleep sessions with same number of clusters. **f** Representative examples of clusters (k-means range 2 to 60) in shuffled sleep datasets in a cuboid- (left) and sphere-reared (right) rat. **g** Shuffled datasets for Day1 sleeps had significantly higher clusters than physiological neural data (p=0.0020, p=0.0020, two-sided signed-rank tests, for comparison of data vs. shuffles in both groups at k-means range 2 to 60). The number of clusters were similar in both shuffled groups (p=0.94, two-sided rank-sum test). **h** Neuronal ensemble similarity scores were significantly, but marginally, lower in cuboid-reared adults compared to cuboid-reared P23-24 rats ($p=6.5x10^{-5}$, two-sided rank-sum test). **i** (Left) Distinct ensembles of neuronal activity were observed in Pre-Day1Run1 sleep of adult cuboid-reared animals. (Right) Similar number of clusters were observed in cuboid-reared P23-24 and adult rats (p=0.96, two-sided rank-sum test). **j** Experience of the linear track during Day1Run1 reduced proportion of significant frames in cuboid- and sphere-reared rats, with a larger decrease observed in sphere-reared rats (two-sided Z-test for 2 proportions). N (rats): Cuboid, P23-24 (5); Sphere, P24 (5). (h-i) N (rats): Cuboid, P23-24 (5), adult (3). Data are displayed as mean±standard error of the mean. ***p<0.005. ns=not significant.

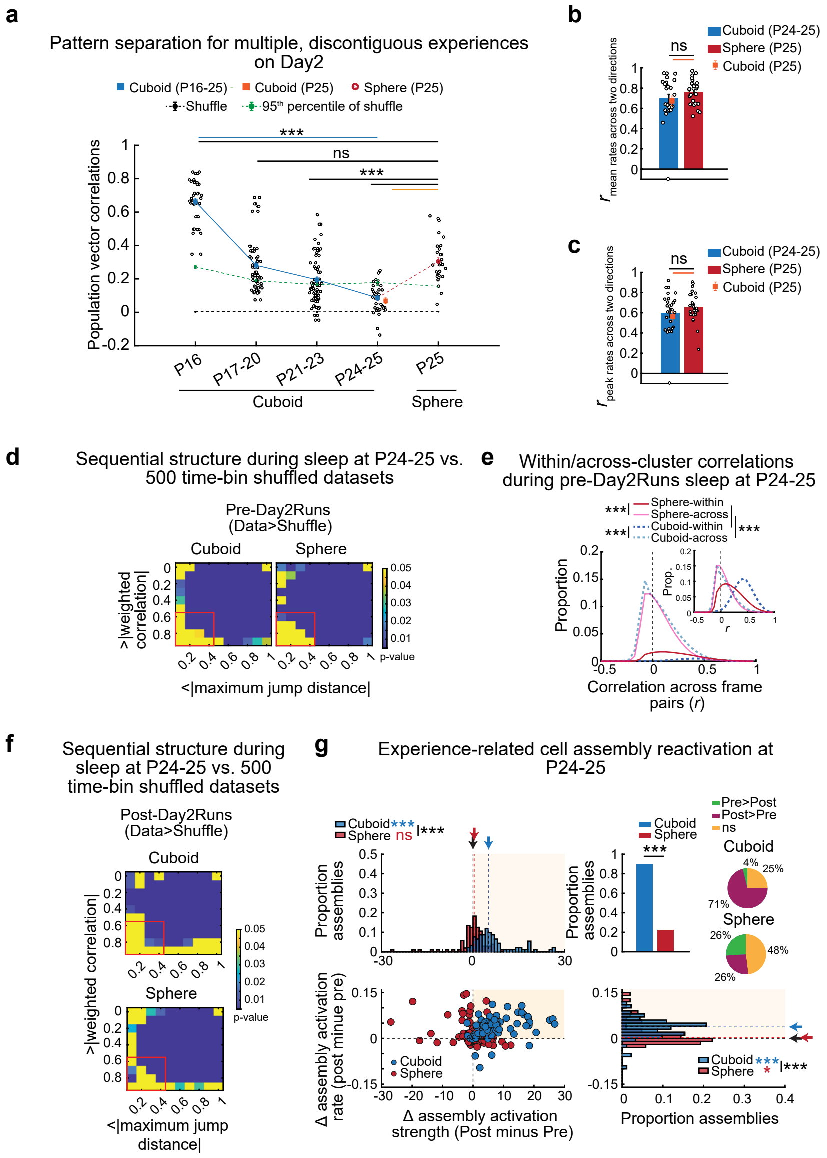

Supplementary Fig. 6: Properties of recruited neuronal ensembles and reactivation for multiple, simultaneous linear track experiences on Day2 in cuboid- and sphere-reared rats. a Age-related increase in pattern-separation (re/mapping) in P16-P25 cuboid-reared rats for the two discontiguous tracks (i.e., tracks 2 and 3) on Day2 ($p=6.8\times10^{-48}$, ANOVA) and its impairment in P25 sphere-reared rats (P25 sphere vs. P16 cuboid: $p=1.1\times10^{-11}$; P25 sphere vs. P17-20 cuboid: p=0.11; P25 sphere vs. P21-23 cuboid: p=0.00012, P25 sphere vs. P24-25 cuboid: $p=6.2\times10^{-10}$, P25 sphere vs. P25 cuboid: $p=2.5\times10^{-9}$, two-sided rank-sum tests). N (rats/group x 2 directions/rat for each track): Cuboid, 6 (P16), 10 (P17-20), 6 (P21-23), 10 (P24-25), 8 (P25); Sphere: 10 (P25). **b-c** Mean (b) and peak (c) firing rates across the two directions of travel were similarly correlated in cuboid- and sphere-reared animals (p=0.29 and p=0.38, two-sided rank-sum tests). Orange marker indicates average values and comparisons with P25 cuboid-reared rats only (p=0.13 and p=0.10, two-sided rank-sum tests). **d** Cuboid- and sphere-reared rats exhibited higher sequential structure (as assessed by simultaneous 2-feature - absolute weighted correlation and maximum jump distance - comparisons of trajectory sequences) than the within-frame time-bin shuffled datasets in the Pre-Day2Runs sleep sessions for novel tracks 2 and 3. Red rectangle depicts sets of thresholds with better sequential content as assessed by these features. Note that multiple 2-feature thresholds are significant (blue) within the red rectangle. **e** Distributions of Pre-Day2Run1 sleep frame-pair correlations (within>across clusters, p=0; sphere vs. cuboid, within or across clusters: p=0, exact tests). Inset: normalized within- and across-cluster correlation values. **f** Cuboid- and sphere-reared rats exhibited higher sequential structure (as assessed by simultaneous 2-feature - absolute weighted correlation and maximum jump distance - comparisons of trajectory sequences) than the within-frame time-bin shuffled datasets in the Post-Day2Runs sleep sessions for novel tracks 2 and 3. Red rectangle depicts sets of thresholds with better sequential content as assessed by these features. Note multiple 2-feature thresholds are significant (blue) within the red rectangle. **g** Reduced cell assembly reactivation (plasticity) during Post-Day2Run2 sleep after sphere-rearing at P25 compared to cuboid-reared rats at P24-25. Scatterplot: Changes in cell-assembly activation strength and rate from Pre-Day2Run1 to Post-Day2Run2 sleep (Strength, Pre vs. Post: cuboid $p=3.0\times10^{-14}$, sphere p=0.15, two-sided signed-rank tests; cuboid vs. sphere: $p=9.7\times10^{-20}$, two-sided rank-sum test; Rate, Pre vs. Post: cuboid $p=4.8\times10^{-11}$, sphere p=0.021, two-sided signed-rank tests; cuboid vs. sphere: $p=6.4\times10^{-11}$, two-sided rank-sum test). Histograms: difference between Pre-Day2Run1 and Post-Day2Run2 sleep in individual features (cell assembly activation strength or rate). Shaded quadrant: assemblies with higher strength and rate in Post-Day2Run2 sleep. Higher proportion of assemblies with both higher strength and rate (top-center, $p=1.0\times10^{-20}$, two-sided Z-test for 2 proportions), and individual plastic assemblies after cuboid- compared to sphere-rearing (top-right, pie-charts, using a rank-sum test on each assembly). (b-g) N (rats/group x 2 directions/rat for each track): Cuboid, P24-25 (10), Sphere, P25 (10). Data are displayed as mean±standard error of the mean. Dashed lines depict medians. ***p<0.005. *p<0.05. ns=not significant.

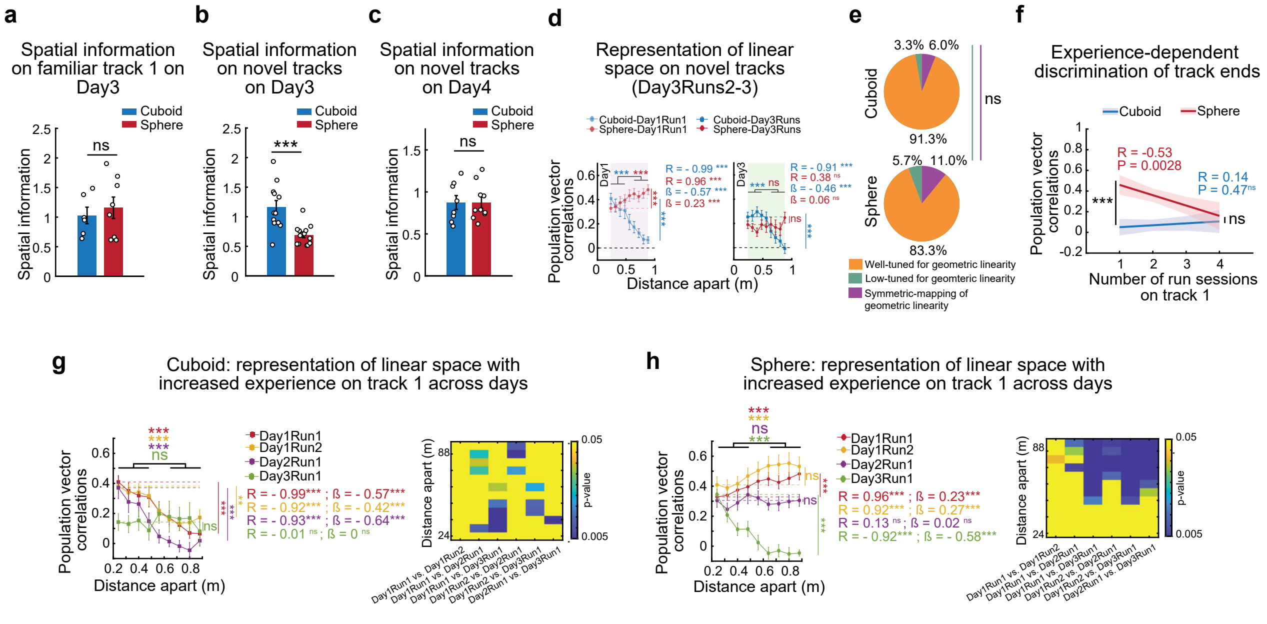

Supplementary Fig. 7: Role of experience with geometric linearity across four days in the development of spatial representation in sphere- vs. cuboid-reared rats. a Spatial information on familiar track 1 (4th run session on track 1 across days) was similar across groups on Day3 (p=0.75, two-sided rank-sum test). **b** Spatial information on novel tracks was significantly lower in sphere- vs. cuboid-reared rats on Day3 (p=0.00075, two-sided rank-sum test). **c** Spatial information on novel tracks was similar across groups on Day4 (p=0.90, two-sided rank-sum test). **d** Population vector correlations between place maps at symmetrical locations on the novel tracks as a function of distance between the corresponding locations during Day3Runs2-3 (right) and Day1Run1 (left). Pearson's correlations and slopes for the regression line fits (R and ß stars mark significance as below) between population vector correlations and distance were computed for the ranges marked by colored masks. Significant increase in population vector correlations in sphere-reared rats (left) as distance increases on Day1, but not on Day3 (closest vs. furthest location on Day3 - 0.24 vs. 0.88 - cuboid: p=1.6×10^{-4}, sphere: p=0.45, two-sided signed-rank tests, depicted by stars on right of plot; four spatial locations in middle vs. four spatial locations at ends, cuboid: p=1.2×10^{-7}, sphere: p=0.94, two-sided rank-sum tests, depicted towards top of plot). **e** Pie-charts depicting proportion of individual rate maps well-tuned for novel linear space, exhibiting symmetric rate maps across the linear track and exhibiting low tuning for linear space. Rate maps with symmetric track firing and low tuning for linear space did not occur significantly more frequently in sphere-reared rats compared with cuboid-reared rats (p=0.075 and p=0.14, two-sided Z-tests for 2 proportions). **f** Network discrimination of track ends improved when rats run on the same track across multiple days (2 exposures on Day1, 1 each on Days2-3) selectively in the sphere-reared rats (Pearson's correlations), which became similar to cuboid-reared rats by the 4th exposure on Day3 (p=0.49, two-sided rank-sum test). Shaded regions are 95% confidence intervals. **g-h** Comparison of population vectors between pairs of place maps at equal distances from the 2 track ends as a function of distance between the corresponding locations across multiple exposures to track 1 across days in cuboid-reared (g, left) and sphere-reared rats (h, left). P-value matrix for comparisons of population vectors at various locations between runs of varying experience of the same track (right; rank-sum tests). For (g-h), Pearson's correlations and ß-coefficients for the regression line fits (R and ß stars mark significance as below) between population vector correlations and distance were computed. Note that while correlations between population vector correlations and distance in cuboid-reared rats (g, left) are significantly negative (on Days1-2Runs) or non-significant negative (on Day3Run1 owing to lower population vector correlations at lower distances potentially due to improved spatial tuning), a shift in significant positive (i.e., the altered representation of the linear track) to negative correlations was observed in sphere-reared rats on increased experience with geometric linearity over days. N (rats/group x 2 directions/rat for each track) on Day3: Cuboid-reared=6; Sphere-reared=8. N (rats/group x 2 directions/rat for each track) on Day4: Cuboid-reared=6; Sphere-reared=8. Data are displayed as mean±standard error of the mean. ***p<0.005. **p<0.01. ns=not significant.

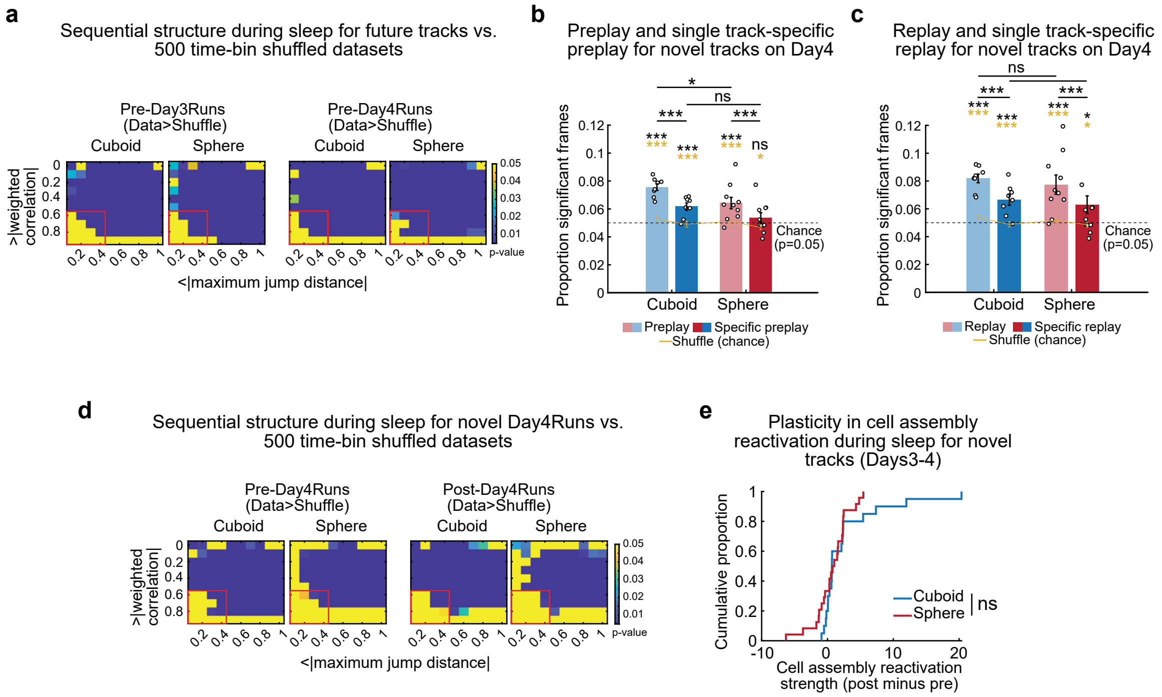

Supplementary Fig. 8: Role of experience with geometric linearity across four days in the reshaping of neuronal sequences in sphere- vs. cuboid-reared rats. a Cuboid- and sphere-reared rats exhibited higher sequential structure (as assessed by simultaneous 2-feature - absolute weighted correlation and maximum jump distance - comparisons of trajectory sequences) than the within-frame time-bin shuffled datasets in the Pre-Day3Runs and Pre-Day4Runs sleep sessions for future tracks. Red rectangle depicts sets of thresholds with better sequential content as assessed by these features. Note that multiple 2-feature thresholds are significant (blue) within the red rectangle. **b** Incidence of preplay for novel tracks was significantly above chance levels in both groups (left, cuboid vs. shuffle: $p=6.4\times10^{-5}$, sphere vs. shuffle: $p=7.5\times10^{-4}$, paired one-sided t-tests, yellow stars; cuboid vs. p=0.05 chance: $p=6.3\times10^{-6}$, sphere vs. p=0.05 chance: p=0.0037, one-sided t-tests, black stars; p=0 and $p=4.4\times10^{-16}$, one-sided Binomial tests vs. p=0.05 chance) and was marginally but significantly lower in sphere-reared rats on Day4 (p=0.040, two-sided t-test, left). Incidence of track-specific preplay for novel tracks was above chance levels in both groups (cuboid vs. shuffle: p=0.0016, sphere vs. shuffle: p=0.014, paired one-sided t-tests; cuboid vs. p=0.05 chance: p=0.0015, sphere vs. p=0.05 chance: p=0.16, one-sided t-tests, black stars; $p=1.0\times10^{-7}$, p=0.0035, one-sided Binomial tests vs. p=0.05 chance) and was similar in both groups on Day4 (p=0.10, two-sided t-test). **c** Incidence of replay for novel tracks was significantly above chance levels in both groups (left, cuboid vs. shuffle: $p=1.5\times10^{-5}$, sphere vs. shuffle: p=0.0018, paired one-sided t-tests, yellow stars; cuboid vs. p=0.05 chance: $p=9.0\times10^{-6}$, sphere vs. p=0.05 chance: p=0.0016, one-sided t-tests, black stars; p=0 for both groups, one-sided Binomial tests vs. p=0.05 chance) and similar in both groups on Day4 (p=0.60, two-sided t-test, left). Incidence of track-specific replay for novel tracks was above chance levels in both groups (cuboid vs. shuffle: $p=4.0\times10^{-4}$, sphere vs. shuffle: p=0.018, paired one-sided t-tests; cuboid vs. p=0.05 chance: p=0.0021, sphere vs. p=0.05 chance: p=0.036, one-sided t-tests, black stars; $p=8.3\times10^{-14}$, $p=3.4\times10^{-5}$, one-sided Binomial tests vs. p=0.05 chance) and similar in both groups on Day4 (p=0.66, two-sided t-test). **d** Cuboid- and sphere-reared rats exhibited higher sequential structure (as assessed by simultaneous 2-feature - absolute weighted correlation and maximum jump distance - comparisons of trajectory sequences) than the within-frame time-bin shuffled datasets in the Pre-Day4Runs and Post-Day4Runs sleep sessions for novel tracks. Red rectangle depicts sets of thresholds with better sequential content as assessed by these features. Note that multiple 2-feature thresholds were significant (blue) within the red rectangle. **e** Cell-assembly reactivation during Post-Day3-4Runs sleep for novel tracks. Plasticity in reactivation was similar in cuboid- and sphere-reared rats (p=0.44, two-sided rank-sum test). N (rats/group x 2 directions/rat for each track) on Day3: Cuboid-reared=6; Sphere-reared=8. N (rats/group x 2 directions/rat for each track) on Day4: Cuboid-reared=6; Sphere-reared=8. Data are displayed as mean±standard error of the mean. ***p<0.005. *p<0.05. ns=not significant.
