# Supplementary material for: Experience of Euclidean geometry sculpts the development and dynamics of rodent hippocampal sequential cell assemblies
Source: Nat Commun. 2024 Sep 28;15:8417. doi: 10.1038/s41467-024-52758-9 (PMC11438871; doi:10.1038/s41467-024-52758-9)
Supplement: Supplementary file 2 — Reporting Summary [file 41467_2024_52758_MOESM2_ESM.pdf]

nature portfolio

Corresponding author(s): George Dragoi

Last updated by author(s): Aug 29, 2024

Reporting Summary

Nature Portfolio wishes to improve the reproducibility of the work that we publish. This form provides structure for consistency and transparency in reporting. For further information on Nature Portfolio policies, see our Editorial Policies and the Editorial Policy Checklist.

Statistics

For all statistical analyses, confirm that the following items are present in the figure legend, table legend, main text, or Methods section.

n/a	Confirmed	
☐	☒	The exact sample size (*n*) for each experimental group/condition, given as a discrete number and unit of measurement
☐	☒	A statement on whether measurements were taken from distinct samples or whether the same sample was measured repeatedly
☐	☒	The statistical test(s) used AND whether they are one- or two-sided<br>*Only common tests should be described solely by name; describe more complex techniques in the Methods section.*
☒	☐	A description of all covariates tested
☐	☒	A description of any assumptions or corrections, such as tests of normality and adjustment for multiple comparisons
☐	☒	A full description of the statistical parameters including central tendency (e.g. means) or other basic estimates (e.g. regression coefficient) AND variation (e.g. standard deviation) or associated estimates of uncertainty (e.g. confidence intervals)
☐	☒	For null hypothesis testing, the test statistic (e.g. F, t, r) with confidence intervals, effect sizes, degrees of freedom and P value noted<br>*Give P values as exact values whenever suitable.*
☐	☒	For Bayesian analysis, information on the choice of priors and Markov chain Monte Carlo settings
☒	☐	For hierarchical and complex designs, identification of the appropriate level for tests and full reporting of outcomes
☐	☒	Estimates of effect sizes (e.g. Cohen's d, Pearson's r), indicating how they were calculated

Our web collection on statistics for biologists contains articles on many of the points above.

Software and code

Policy information about availability of computer code

Data collection	Electrophysiological and behavioral data were collected by Cheetah (Neuralynx), versions 5.6.3 and 5.7.0
Data analysis	Most of the analysis was performed in MATLAB (R2015a, R2020a) or python (3.7). Spike sorting was performed by Xclust3. Codes used in this study are available at: https://github.com/GDYlab/GDYlabcode

For manuscripts utilizing custom algorithms or software that are central to the research but not yet described in published literature, software must be made available to editors and reviewers. We strongly encourage code deposition in a community repository (e.g. GitHub). See the Nature Portfolio guidelines for submitting code & software for further information.

Data

Policy information about availability of data

All manuscripts must include a data availability statement. This statement should provide the following information, where applicable:

- Accession codes, unique identifiers, or web links for publicly available datasets
- A description of any restrictions on data availability
- For clinical datasets or third party data, please ensure that the statement adheres to our policy

Data used in this study are available from the corresponding author upon request. The reported data are archived on file servers at the Yale Medical School. The very large size of the raw data prohibits its public availability. No clinical datasets or genetics data have been used in this study.

Research involving human participants, their data, or biological material

Policy information about studies with human participants or human data. See also policy information about sex, gender (identity/presentation), and sexual orientation and race, ethnicity and racism.

Reporting on sex and gender	N/A
Reporting on race, ethnicity, or other socially relevant groupings	N/A
Population characteristics	N/A
Recruitment	N/A
Ethics oversight	N/A

Note that full information on the approval of the study protocol must also be provided in the manuscript.

Field-specific reporting

Please select the one below that is the best fit for your research. If you are not sure, read the appropriate sections before making your selection.

☒ Life sciences ☐ Behavioural & social sciences ☐ Ecological, evolutionary & environmental sciences

For a reference copy of the document with all sections, see nature.com/documents/nr-reporting-summary-flat.pdf

Life sciences study design

All studies must disclose on these points even when the disclosure is negative.

Sample size	Sample size were indicated for each experiment and were chosen based on similar studies. No statistical methods were used to predetermine sample size, but our sample sizes are similar to those reported in previous publications (Farooq and Dragoi, Science 2019).
Data exclusions	No data were excluded in general. The criteria of each analysis was stated in the description of each analysis.
Replication	The number of times each experiment is repeated is mentioned in the text, methods and figure legends. All experimental findings were reliably reproduced. The sphere vs. age-matched cuboid experiment was performed with 5 animals per group.
Randomization	Data were randomized to construct control groups in individual analysis.
Blinding	Treatment of randomized control groups for individual analysis were described in Methods. Data collection and analysis were not performed blind to the conditions of the experiments.

Reporting for specific materials, systems and methods

We require information from authors about some types of materials, experimental systems and methods used in many studies. Here, indicate whether each material, system or method listed is relevant to your study. If you are not sure if a list item applies to your research, read the appropriate section before selecting a response.

Materials & experimental systems

n/a	Involved in the study	
☐	☒	Antibodies
☒	☐	Eukaryotic cell lines
☒	☐	Palaeontology and archaeology
☐	☒	Animals and other organisms
☒	☐	Clinical data
☒	☐	Dual use research of concern
☒	☐	Plants

Methods

n/a	Involved in the study	
☒	☐	ChIP-seq
☒	☐	Flow cytometry
☒	☐	MRI-based neuroimaging

Antibodies

Antibodies used	A commercial corticosterone ELISA kit (Arbor Assays, catalog no. K014, Ann Arbor, MI, USA) was used for fecal corticosterone

Antibodies used	assessment.
Validation	The kit has been pre-validated extensively for various samples and species including rat fecal matter. Both groups were simultaneously assessed using the same kit following the same procedure.

Animals and other research organisms

Policy information about studies involving animals; ARRIVE guidelines recommended for reporting animal research, and Sex and Gender in Research

Laboratory animals	Long-Evans rats of both sexes with ages starting from postnatal day 15 until adult life, 3-6 months old.
Wild animals	This study did not involve wild animals.
Reporting on sex	Male rats were used for the neurophysiological recordings (partly due to their higher weights facilitating carrying of headstages) and stress quantification. Since intrinsic litter movement dynamics were studied, movement was recorded from the whole litter including the mother and both sex litter mates.
Field-collected samples	This study did not involve field-collected samples.
Ethics oversight	IACUC at Yale University

Note that full information on the approval of the study protocol must also be provided in the manuscript.

Plants

Seed stocks	N/A
Novel plant genotypes	N/A
Authentication	N/A
